# Comparison of the efficacy and safety of tacrolimus monotherapy and cyclophosphamide combined with glucocorticoid in the treatment of adult primary membranous nephropathy: protocol of a multicenter, randomized, controlled, open study

**DOI:** 10.1186/s13063-020-4144-3

**Published:** 2020-02-24

**Authors:** Shasha Chen, Song Ren, Amanda Y. Wang, Hieu Tran, Zheng Li, Xiaoyun Cheng, Manli Liu, Guisen Li, Li Wang, Daqing Hong

**Affiliations:** 1 Renal Division and Institute of Nephrology, Sichuan Academy of Medical Sciences and Sichuan Provincial People’s Hospital, Medical School of University of Electronic Science and Technology of China, Chengdu, 610072 China; 2 Renal and Metabolic Division, The George institute for global health, Sydney, Australia; 3 The Faculty of Medicine and Health Sciences, Macquarie University, Sydney, Australia; 4 Department of Medicine, Ryde Hospital, Sydney, Australia; 5 Renal Department, Chengdu First People’s Hospital, Chengdu, 610041 China; 6 Renal department, Chengdu Second People’s Hospital, Chengdu, 610017 China; 7 Renal department, Chengdu Third People’s Hospital, Chengdu, 610031 China

**Keywords:** Tacrolimus, Cyclophosphamide, Glucocorticoid, Membranous nephropathy, Randomized controlled trial

## Abstract

**Background:**

Idiopathic membranous nephropathy (IMN) remains the leading cause of adult nephrotic syndrome. Immunosuppressive therapy with cyclophosphamide (CTX) is often successful in reducing proteinuria, but its use is associated with severe side effects. Tacrolimus (TAC) is effective in achieving complete remission (CR) in patients with IMN. However, whether it is as effective as CTX in inducing and maintaining complete or partial remission in these patients is unknown. This trial aims to test TAC monotherapy for its non-inferiority to CTX in inducing long-term remission of proteinuria.

**Methods:**

Patients with biopsy-proven IMN with nephrotic syndrome will be randomized into a 12-month treatment period with oral TAC of 0.05–0.1 mg/kg/day for 6 months or with CTX + glucocorticoid. The efficacy of the treatment will be assessed by the remission status (based on changes in proteinuria) and relapse rate.

**Discussion:**

This study will test whether treatment with TAC monotherapy is superior to CTX with glucocorticoid in inducing long-term remission of proteinuria in patients with adult IMN. The role of serum anti-PLA2R antibodies in the early assessment of the response to therapy using different therapeutic regimens will also be clarified.

**Trial registration:**

ClinicalTrials.gov ChiCTR1800016140. Registered 12 June 2017. http://www.chictr.org.cn.

## Background

Idiopathic membranous nephropathy (IMN) is one of the most common pathological types of primary nephrotic syndrome in adults [1]. The peak age is 40 to 50 years old, and IMN accounts for approximately 13.3% of the primary glomerular disease [2] and 20.7% of the nephrotic syndrome in China [3]. Membranous nephropathy (MN) shows a benign or indolent course in some patients, with a rate of spontaneous complete or partial remission as high as 30% or more [4, 5]. Despite this, 30% to 40% of patients progress toward end-stage renal disease (ESRD) within 5 to 15 years [4, 6]. The KDIGO (Kidney Disease: Improving Global Outcomes) guidelines recommend steroids combined with alkylating agents in patients with severe proteinuria who are at high risk of developing ESRD. Glucocorticoid combined with CTX (cyclophosphamide) is currently the most commonly used treatment option in clinical practice and is also the initial treatment regimen recommended by the KDIGO guidelines [7]. A large number of clinical trials have shown that glucocorticoid combined with CTX can effectively reduce proteinuria, protect renal function, and reduce the risk of death or end-stage renal disease [8–10]. The administration methods of CTX include oral and intravenous administration. The total remission rate of intravenous administration is 73–87% [11, 12], oral administration is 64–88% [10, 12, 13], and no significant difference exists between the two methods, but intravenous administration has the following advantages over oral administration: 1) Before intravenous infusion, the patients need to check blood routine, liver and kidney function, and other related indicators, and cyclophosphamide can be given only when the indicators allow, thus ensuring the safety of patients and possibly reducing the risk of serious adverse effects. 2) The doctors can more accurately understand the cumulative dose of cyclophosphamide, thereby avoiding the impact of some patients on the study due to irregular oral medication or irregular follow-up. 3) According to our experience, oral cyclophosphamide has a faster cumulative dose and shows greater adverse reactions including leukopenia, myelosuppression, and infection than intravenous infusion. Based on the above points, We chose intravenous rather than oral cyclophosphamide. TAC has also been widely used in IMN. Most studies have shown that TAC alone or combined with low-dose glucocorticoids has achieved good outcomes [14–16].

We used network meta-analysis to directly and indirectly compare the efficacy of different immunosuppressive regimens on renal survival, proteinuria remission, and tolerability in IMN. Our review showed that CTX and chlorambucil reduced the risk of ESRD or death in IMN with nephrotic range proteinuria but carry substantial toxicity that may be lower for CTX [17], whereas contemporary evaluations of the side effects of cyclophosphamide show far fewer serious side effects [18–20]. In comparison, tacrolimus (TAC) and cyclosporine decreased the possibility of proteinuria relapse after drug withdrawal, the effects on renal impairment of TAC remained uncertain [17]. Monotherapy with TAC was shown to have a similar effect on proteinuria remission with fewer side effects, which were predominately caused by steroids [15, 21]. These studies, however, were relatively small and underpowered.

The purpose of this study is to verify that the efficacy of TAC alone is not inferior to CTX combined with corticosteroids in reducing proteinuria. The secondary objectives are to evaluate whether TAC alone reduces the number of relapses (efficacy in sustaining remission) and increases the time to relapse when compared with treatment with CTX, as TAC alone is supposed to have a better side-effect profile and improve the quality of life when compared with treatment with CTX in patients with IMN. Serum anti-PLA2R levels at 6, 12, and 24 months post-treatment compared with baseline and its correlation with clinical parameters and therapeutic effectiveness will also be investigated.

## Methods/Design

### Design

This is a prospective, randomized, controlled, open, clinical trial. The registration number is ChiCTR1800016140. The protocol was Version 2.0, which was revised on 22 January 2018. The data safety and monitoring board (DSMB) provided the study monitoring, randomization, data management, safety surveillance, and biostatistics services.

### Study setting

The study will be held in four Chinese nephrology centers, including the lead center—Sichuan Provincial People’s Hospital— and the First People’s Hospital of Chengdu, the Second People’s Hospital of Chengdu, and the Third People’s Hospital of Chengdu. The study commenced in December 2018 and will end in June 2021. The data analysis and report will be completed by December 2021. A total of 90 patients will be enrolled in the participating four research centers in Sichuan Province. Each research center in the trial will have at least 25 cases enrolled and have approximately 150 cases screened. The screening failure rate is estimated to be less than 30%, and the loss rate less than 10%.

### Population

MN is histologically characterized by immune deposits on the subepithelial side of the glomerular basement membrane with spike formation by light microscopy, a fine granular distribution of immunoglobulin (Ig) G, and the complement component C3 in a capillary-loop pattern revealed by immunofluorescence, and the presence of electron-dense subepithelial immune deposits indicated by electron microscopy (EM).

Patients with biopsy-proven IMN manifested as nephrotic syndrome will be screened.

Serum autoantibodies to the M-type phospholipase A2 receptor (anti-PLA2R antibodies) (ELISA, Euroimmun AG, Luebeck, Germany) and PLA2R antigen staining on renal tissues at baseline in all selected participants will be recorded. Each participating center will collect blood samples for these determinations at Months 0, 4, 12, 24, and 48 and optionally at Months 3, 9, and 18. Blood samples for these special determinations shall be kept in each participating center at -80 °C.

### Eligibility criteria

#### Inclusion and exclusion criteria

Inclusion and exclusion criteria are listed in Table 1.

**Table 1:** Inclusion and exclusion trial criteria for patients

Inclusion criteria	Exclusion criteria
-Written and informed consent will be obtained. -Age 18–65 years	-Secondary membranous nephropathy (e.g., hepatitis B, SLE, medications, malignancies, etc.)
-Urinary protein excretion persistently exceeds 3.5 g/d, serum albumin < 30 g/L) after 6 months of antiproteinuric therapy with ACEI/ARB -IMN diagnosed by renal biopsy	-Positive HBV serological indexes (HBsAg or/and HBeAg or HBcAb), positive HCV or patients with abnormal liver function (ALT, AST, or bilirubin show an increase two times higher than the upper limit of normal range for more than 2 weeks)
-Serum creatinine < 133umol/L	-Diagnosis of diabetes or impaired glucose tolerance (2 h post-prandial plasma glucose 7.8–11 mmol/L)
-No immunosuppressive treatment in the previous 6 months	-Have a definite history of peptic ulcer and/or gastrointestinal bleeding within the preceding 6 months -With congenital or acquired immunodeficiency, or with infections such as active tuberculosis and active CMV, or with severe infections requiring intravenous antibiotic therapy -With other serious physical or mental illness -With congenital heart disease, arrhythmia, heart failure and other serious cardiovascular diseases -Pregnancy or inadequate contraception -Have participated in other clinical trials within three months prior to enrollment

#### Exit criteria

The exit criteria are as follows:

1. Voluntary request for withdrawal by participants or their legal guardians
2. Violation of inclusion/exclusion criteria
3. Patients being treated with drugs
4. Occurrence of serious adverse events such that the participant is unable to continue the study, including severe infection and uncontrolled hyperglycemia
5. Patients who, when treated with TAC, show a deterioration of renal function, manifested as SCr increase to ≥ 30% of the baseline value, and SCr > 133 umol/L (excluding non-drug related factors). TAC dosing should then be reduced by 30% with weekly review of renal function. Participants with SCr rising to ≥ 50% of the base value after 2 weeks will exit from the study.
6. Participants show ALT, AST, and/or bilirubin elevation two times higher than the normal range. Patients with ALT (alanine aminotransferase), AST (glutamic-oxaloacetic transaminase), and/or bilirubin elevation two times higher than the upper limit should be treated with hepatoprotective medicine for 2 weeks. The participant will then exit from the study.
7. Occurrence of pregnancy during the treatment
8. Study stopped by study sponsor based on the advice of the Data Safety Monitoring Board

The exit rate will be calculated at the end of the study. A detailed record for the reason and date of the participant’s withdrawal will be kept. Participants who withdraw from the study will be given appropriate treatment based on the judgment of the researchers at the participating centers, and follow-up of their treatment effects will continue.

### Discontinuation

The intervention may be discontinued if the investigator decides to discontinue the intervention due to adverse event(s) or any other reason or if the participant requests discontinuation.

### Randomization

We first generate a random code by computer; then, we create a random envelope according to the random code, and the eligible patients are randomized into TAC monotherapy or steroids plus CTX group in a random ratio of 1:1. Participant numbers will be assigned sequentially as each patient enters the study.

### Treatment regimen (Fig. 1)

#### Intervention group

1. Initial treatment:

**Fig. 1:**
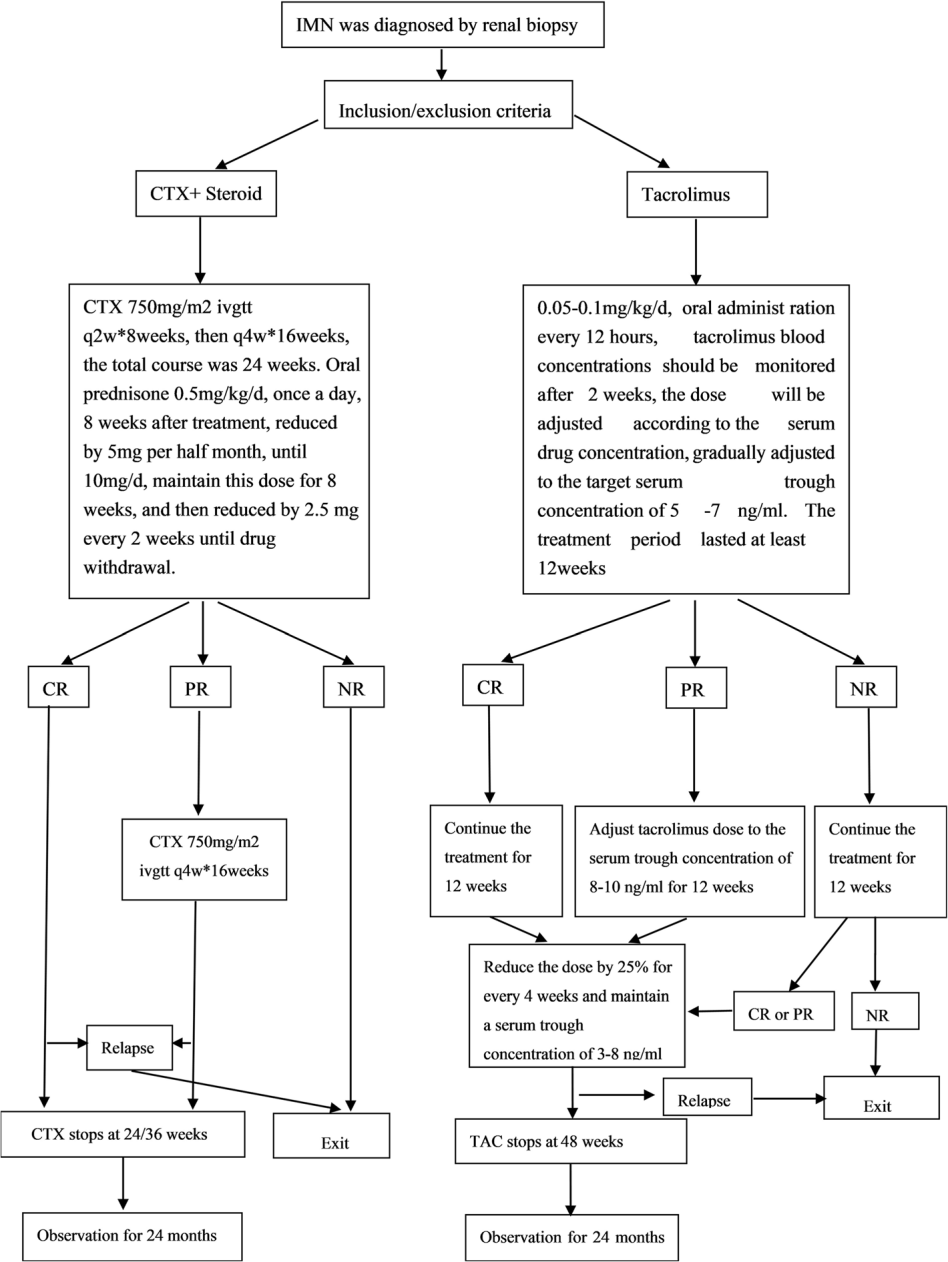
Schematic representation of the study design therapy

The TAC treatment group will be initiated on a starting dose of 0.05–0.1 mg/kg/day p.o., divided into two equal doses given at 12-h intervals. The dose is adjusted according to the target trough blood concentration of 5–7 ng/ml. TAC dosage should be reduced by 30% when a 30% increase in serum creatinine is noted compared with the baseline value, and TAC is withdrawn if the renal function is not improved after 2 weeks. This treatment period is for at least 12 weeks.

2) Maintenance phase treatment:

A) Complete remission (CR)—Patients with CR after 12 weeks will continue to use the original treatment program to 24 weeks. The dosage of TAC will be reduced at intervals of 4–8 weeks to a level of approximately 50% of the starting dosage, provided that remission is maintained and no treatment-limiting CNI-related nephrotoxicity occurs, and this dosage will be continued for at least 12 months.
B) Partial remission (PR)—Patients with PR after 12 weeks are recommended to adjust TAC dosage to the serum trough concentration of 8–10 ng/ml, and after 24 weeks, the dose will be gradually reduced (same method as CR patients).

3) Invalid response or patient relapse:
  Participants will be withdrawn from the study if they have no remission or experience a relapse of proteinuria after 24 weeks of TAC treatment, and then they will continue with other therapy.
4) Course of treatment:
  The total course of treatment is approximately 48 weeks. The total observation course is approximately 24 months.

### Control group

The control group will be managed as follows:

1. The patients in the control group will be treated with CTX combined with corticosteroids [13], with intravenous infusion of cyclophosphamide being administered at 750 mg/m2/2 weeks for 8 weeks and then every 4 weeks for the next 16 weeks (8 pulses in total). Oral prednisone will be administered at a dose of 1 mg/kg/d for 4 weeks, tapering to 5 mg every 2 weeks to 30 mg/d and then being reduced by 5 mg every 4 weeks until complete withdrawal at the end of 12 months.
2. CR—Cyclophosphamide will be discontinued after 24 weeks of treatment and will be followed up to 24 months.
3. PR—Cyclophosphamide can be given once every 4 weeks for another 12 weeks after the end of the 24-week treatment course. The cumulative course of treatment will be 36 weeks. Thereafter, discontinuation of all drug treatment and will be followed up to the 24th month.
4. Invalid response or relapse—Patients who fail after 24 weeks of treatment or who relapse will withdraw from the study.
5. Administration of glucocorticoid—Oral prednisone of 0.5 mg/kg/d, 8 weeks after treatment, will be reduced by 5 mg per half month, until 10 mg/d after 8 weeks of treatment. The dosage will be maintained for 8 weeks and then will be decreased by 2.5 mg every 2 weeks until the drug is completely discontinued. The course of treatment is approximately 24–30 weeks.

### Other treatment

1. Angiotensin-converting enzyme inhibitors/*angiotensin* receptor antagonist (ACEI/ARB): Patients with persistent hypertension can be prescribed for ACEI/ARB, dihydropyridine class calcium channel blockers, beta blockers, diuretics, and/or alpha receptor blockers to target blood pressure < 130/80 mmHg. But the class of choice is ACEI/ARB therapy.
2. Lipid-lowering therapy: Statin class drugs may be used in the study to lower the serum lipid levels.
3. Anticoagulant therapy: Anticoagulants are permissible and can be prescribed according to the clinical scenario during the study.

### Excluded drugs

1. Other immunosuppressive agents are excluded in this study.
2. Immunoglobulins, plasma exchange, and antibodies are also excluded from treatment.
3. Drugs that may interact with TAC (except for essential drugs in the study) are also excluded.
4. Traditional Chinese medicines such as Tripterygium wilfordii and Huangkui capsules are excluded.

### Outcomes

#### Main outcomes

Main outcomes include the following:

1. CR rate—CR rate at 3, 6, 9, 12, and 24 months. CR is defined as a reduction in urine protein to less than 0.3 g/d and an albumin to creatinine ratio of < 30 mg/mmol.
2. PR rate—PR rate at 3, 6, 9, 12, and 24 months. PR is defined as a urine protein decrease to 0.3–3.5 g/d, with a decrease of more than 50% compared with baseline.
3. Relapse rate—The relapse rate of recurrence is defined as urine protein 2+ for more than 3 consecutive days after CR or PR.

#### Secondary outcomes

Secondary outcomes include the following:

1. The percentage of patients who withdraw due to intolerance of adverse drug reactions
2. The proportion of patients whose treatment is ineffective or discontinue and the number of patients who convert to other immunosuppressants
3. Changes in renal function
4. Changes in proteinuria
5. The time of proteinuria remission
6. Changes in serum albumin
7. Creatinine increases of > 40%
8. Serum anti-PLA2R levels before treatment and at 12 and 24 months post-therapy
9. The proportion of patients with ESRD or Death
10. Proportion of patients with drug-related adverse events including amenorrhea, diabetes, and infections during the study

#### Participant timeline

This is a randomized controlled trial with three stages: screening and recruitment of patients (6–12 months), treatment period (9 months for corticosteroids and CTX group and 12 months for TAC), and a post-treatment follow-up period of 24 months from initial treatment.

### Adverse events (AEs) and serious adverse events (SAEs)

Adverse events should be observed and recorded during each follow-up visit. The severity of adverse events and their relationships with the relevant drugs should be evaluated. Long-term follow-up of all patients, including prognosis and mortality outcomes, will be analyzed until the end of the study period in June 2020.

### Sample size calculation

Randomization was carried out using computer-generated simple random tables at 1:1 ratio with non-inferior effect based on the assumption of 60% vs 80% PR + CR rate in the CTX vs TAC group. The study required an estimated 90 participants for an alpha of 0.05 (two-tailed test), a power (1-β) of 0.80, a 10% non-inferior effect difference, and an allowed dropout rate of 10%.

### Research steps

All the patients who sign the informed consent are assigned to a 4-digit number, and this number is composed of a 2-digit center number + 2 digit screening sequence number. Once all the results show that the patients are within the standard of inclusion criteria, random envelopes are split into random numbers, which are subsequently divided into the TAC group or the control group. Efficacy and safety are evaluated at 1, 3, 6, 9, 12, and 24 months after screening. Day 1 is defined as the date of first use of cyclophosphamide or TAC, and all the ensuing follow-up visits are calculated relative to Day 1(Table 2).

**Table 2:** Scheme of the activities that will take place at each contact with the participant after randomization

Visiting	1	2	3	4	5	6	7	8	9	10	11	12	13	14	15	16	17	18
Project	Screening −14~ − 1	D 0	1 w ± 3 D	2 w ± 3 D	4 w ± 3 D	8 w ± 3 D	12 w ± 7 D	16 w ± 7 D	20 w ± 7 D	24 w ± 7 D	28 w ± 7 D	32 w ± 7 D	36 w ± 7 D	40 w ± 7 D	48 w ± 7 D	64 w ± 7 D	80 w ± 7 D	96 w ± 7 D
Informed Consent	X																	
Inclusion/exclusion criteria	X																	
Pregnancy test for female patients	X																	
Randomization		X																
General information	X																	
Physical examination	X	⊙	X	X	X	X	X	X	X	X	X	X	X	X	X	X	X	X
Renal biopsy	X												⊙					
Evaluation of clinical response			X	X	X	X	X	X	X	X	X	X	X	X	X	X	X	X
Urine routine	X		X	X	X	X	X	X	X	X	X	X	X	X	X	X	X	X
24 h-UP, UPCR	X	⊙	X	X	X	X	X	X	X	X	X	X	X	X	X	X	X	X
Routine blood test	X	⊙	X	X	X	X	X	X	X	X	X	X	X	X	X	X	X	X
Blood biochemical index	X	⊙	X	X	X	X	X	X	X	X	X	X	X	X	X	X	X	X
SCr/eGFR	X	⊙	X	X	X	X	X	X	X	X	X	X	X	X	X	X	X	X
Serum anti-PLA2R		X			X		X			X			X		X			X
Stool routine +OB	X												X					
HbA1c	X						X			X			X					
body mass index (BMI)	X						X			X			X			X	Fig	X
Centripetal obesity index (COI)	X						X			X			X			X		X
Mental and psychological status assessment	X						X			X			X			X		X
Quality of life assessment (SF-36 scale)	X						X			X			X			X		X
ECG/Chest X-ray	X												X					
Tacrolimus blood concentration			X	X	X	X	X			X			X					
Specimen of blood and urine	X						X			X			X			X		
Drug dose adjustment			X	X	X	X	X	X	X	X	X	X	X	X	X			
Drug delivery and counting			X	X	X	X	X	X	X	X	X	X	X	X	X			
Combined use of drugs	X	⊙	X	X	X	X	X	X	X	X	X	X	X	X	X	X		
Adverse event		⊙	X	X	X	X	X	X	X	X	X	X	X	X	X	X	X	X
Extra hospitalization							X			X			X			X	X	X

### Statistical analysis

The purpose of this study is to evaluate the efficacy and safety of the two different treatment regimens. In the statistical analysis, the laboratory test results of the evaluation indexes of the screening period should be selected as the baseline value. This study will also carry out sensitivity analysis based on pathological stages, sex, age groups, and so on.

The main outcomes are the remission rates of proteinuria (including CR and PR) and relapse rate at the 12th week, 24th week, and end of the treatment period. The difference in the efficacy of the TAC group with the control group is evaluated by chi-square testing. The Kaplan-Meier method is used to analyze the CR, PR, and relapse rates of the two groups.

Chi square test or Fisher exact probability testing are used to analyze the difference between the two groups for secondary outcomes. For continuous variables (such as eGFR change), the GEE model is compared with repeated measurement data statistics. Chi square test or Fisher exact probability testing are used to analyze the incidence of adverse events in the two groups.

According to the data distribution, the changes of 24-h quantitative urine protein, urinary protein/creatinine, serum albumin, serum creatinine, eGFR, blood glucose, blood lipid, blood uric acid, weight, and blood pressure at baseline used is paired *t* test or Wilcoxon. This method is the rank sum test to intragroup comparison. The two-sample independent t test or non-parametric test is used for intergroup comparison.

## Discussion

KDIGO guidelines recommend that corticosteroids and alkylating agents (cyclophosphamide or chlorambucil) be used for 6 months for MN patients with persistent nephrotic syndrome after 6–12 months of conservative therapy or with a decreased baseline renal function [22]. The percentage of remissions after 1 year of treatment is 50–60%. However, serious adverse effects of this standard therapy, including serious infection, myelosuppression, and gonado-inhibitory effects, limited their clinical applications [23, 24]. The number and severity of side effects are important drawbacks of this therapy.

Recent reports and systematic review demonstrate that calcineurin inhibitors (CNIs) are promising alternatives to CTX for IMN patients, primarily due to their better short-term efficacy and safety profile. A number of randomized controlled trials (RCTs) have reported that TAC combined with corticosteroids demonstrated a satisfactory effect compared with CTX plus corticosteroids [24–26]. However, corticosteroids can still cause adverse effects, such as infection, avascular necrosis of the femoral head, central obesity, and liver dysfunction.

TAC monotherapy is an effective and safe option for the treatment of MN with stable renal function. In 2007, Praga et al. found that TAC monotherapy was effective in the treatment of IMN, increasing the probability of remission in 82% of patients after 12 months of treatment compared with 24% in the control group [15]. Despite the encouraging results of TAC, potential nephrotoxicity and high relapse rate after drug discontinuation are still of concerns [27]. Relapses are frequent in patients with PR and can be partially prevented by a longer tapering period. Currently, little evidence exists to show that TAC could alter the definite endpoints such as all-cause mortality or risk of ESRD. Also, little evidence exists to support the use of TAC monotherapy in MN. This trial is designed to test the hypothesis that TAC alone is superior to cyclophosphamide with glucocorticoid in inducing long-term remission of proteinuria and is safer than alkylating agent combined with corticosteroids to treat IMN.

Therefore, this prospective, randomized, controlled, open, clinical trial will provide significant information regarding the treatment of patients with IMN and provide insight into the risks and benefits of TAC monotherapy in IMN.

## Trial status

Protocol version number and date: Version 2, dated 22 January 2018.

Recruitment start date: 1 December 2018.Expected recruited completion date: 31 June 2021.

## Data Availability

Not applicable at this stage. The datasets analyzed during the current study will be available from the corresponding author on reasonable request when the study is finished.
